# Occurrence of *Listeria monocytogenes* in Artisanal Cheeses from Poland and Its Identification by MALDI-TOF MS

**DOI:** 10.3390/pathogens10060632

**Published:** 2021-05-21

**Authors:** Renata Pyz-Łukasik, Michał Gondek, Dagmara Winiarczyk, Katarzyna Michalak, Waldemar Paszkiewicz, Anna Piróg-Komorowska, Agata Policht, Monika Ziomek

**Affiliations:** 1 Department of Food Hygiene of Animal Origin, Faculty of Veterinary Medicine, University of Life Sciences in Lublin, Akademicka, 12, 20-033 Lublin, Poland; michal.gondek@up.lublin.pl (M.G.); waldemar.paszkiewicz@up.lublin.pl (W.P.); monika.ziomek@up.lublin.pl (M.Z.); 2 Department and Clinic of Animal Internal Diseases, Faculty of Veterinary Medicine, University of Life Sciences, Głęboka, 30, 20-612 Lublin, Poland; winiarczykdm@gmail.com; 3 Department of Epizootiology and Clinic of Infectious Diseases, Faculty of Veterinary Medicine, University of Life Sciences in Lublin, Głęboka, 30, 20-612 Lublin, Poland; kat.michalak86@gmail.com; 4 Department of Veterinary Hygiene, Provincial Veterinary Inspectorate in Krakow, Brodowicza, 13b, 30-965 Kraków 69, Poland; lobo@wiw.krakow.pl (A.P.-K.); agata.policht@wiw.krakow.pl (A.P.)

**Keywords:** *Listeria monocytogenes*, artisanal cheeses, MALDI-TOF MS, food safety

## Abstract

*Listeria monocytogenes* is a foodborne pathogen. A source of infection can be artisanal cheeses. Identification of the *Listeria* species is important for the protection of public health and the food industry. This study aimed to examine artisanal cheeses for the presence of *L. monocytogenes* and the effectiveness of the MALDI-TOF MS method in the identification of the *L. monocytogenes* isolates. A total of 370 samples of artisanal cheeses were examined. *L. monocytogenes* was found in 23 cheese samples (6.2%). The reliability of *L. monocytogenes* identification achieved by MALDI-TOF MS was varied, and the vast majority of the isolates (27/32) were identified only to the secure genus, probable species level. This study showed that (i) the occurrence of *L. monocytogenes* in the artisanal cheeses was at a higher level than that in the other EU countries, (ii) the standard of species identification of *L. monocytogenes* isolates from artisanal cheeses achieved by MALDI-TOF MS was not satisfactory and (iii) the presence of *L. monocytogenes* in artisanal cheeses remains a problem with regard to the food safety criterion and a potential public health risk.

## 1. Introduction

*Listeria monocytogenes* is a real public health threat, as evidenced by the data of zoonosis and zoonotic agent monitoring in European Union (EU) countries. This pathogen is the etiological agent of listeriosis, a foodborne disease. Taking into account the number of reported human cases in the EU in 2018, listeriosis ranks fifth among 13 zoonoses and the highest number of hospitalisations and mortality rates have been noted for this zoonosis. In addition, in recent years (2009–2018), there has been an increase in the number of cases of listeriosis [1].

A source of infection may be artisanal cheeses contaminated with *L. monocytogenes* [2,3,4,5,6,7]. Artisanal cheeses belong to traditional dairy products [8,9,10,11,12], and they are constantly produced in many countries of the world. This type of foodstuff has cultural, social and economic importance [13].

Cheeses should meet the safety criterion concerning *L. monocytogenes*; a product that does not comply with this criterion is considered unsafe and cannot be offered for sale [14,15]. According to the literature, MALDI-TOF MS (matrix-assisted laser desorption ionisation–time of flight mass spectrometry) is considered a reliable, fast and cost-effective tool for the routine identification of *Listeria* [16,17]. This method consists of bacterial protein panel analysis in the mass range of 2–20 kDa, which mainly represents ribosomal proteins and basic metabolism proteins. These proteins create a bacteria-specific fingerprint, which, when compared with the protein profiles contained in a reference spectrum library, enables the determination of the taxonomic position of the microorganism. The MALDI-TOF MS analysis evaluates two parameters: the ion mass-to-charge ratio (m/z) and the relative ion intensity. The protein spectrum obtained in this procedure is processed to yield the protein code of the microorganism. A comparison of the obtained code against the reference codes contained in the library enables the identification of the microorganism at the genus, species, subspecies or strain level [18]. This technique enabled the identification of *L. monocytogenes* with 100% accuracy, regardless of its origin, whether from humans, animals, food or the environment [16,17]. On the other hand, other authors have shown that the MALDI-TOF MS method is not without limitations in identifying *L. monocytogenes* [19].

The objectives of this study were the examination of artisanal cheeses for the presence of *L. monocytogenes* and the evaluation of the usefulness of the MALDI-TOF MS method in the identification of *L. monocytogenes* isolates derived from these types of foodstuffs.

## 2. Results

### 2.1. Bacteriological Analysis and Identification by MALDI-TOF MS

A total of 370 samples of artisanal cheeses were analysed for the presence of *L. monocytogenes*. The pathogen was detected in 23/370 samples of the cheeses (6.2% of all samples tested). The products contaminated with *L. monocytogenes* came from 16 production sites. During the investigation period (2014–2018), the number of cheese dairies from which tested products contained *L. monocytogenes* was between two in 2014 and 2015 and eight in 2018; a rising trend was observed from 2016. A total of 32 isolates of *L. monocytogenes* were yielded: in three cases, three isolates from one batch of tested cheeses were obtained, and the relevant production facilities were G1, I and H; in another three cases, two isolates from one batch of tested cheeses were obtained, and the relevant production facilities were G2, F and I; in the remaining 17 cases, one isolate from one batch of tested cheeses was obtained (see Section 4.1. *Materials* and Table 1).

The score values for the tested isolates by MALDI-TOF MS indicated that one isolate (3.1%) was identified at the level of highly probable species identification (log(score) 2.306–2.368), 27 isolates (84.4%) were identified at the level of secure genus identification and probable species identification (log(score) ≥ 2.0–2.29) and four isolates (12.5%) were identified at the level of probable genus identification (log(score) 1.901–1.997) (Table 1; Tables S1 and S2).

**Figure 1:**
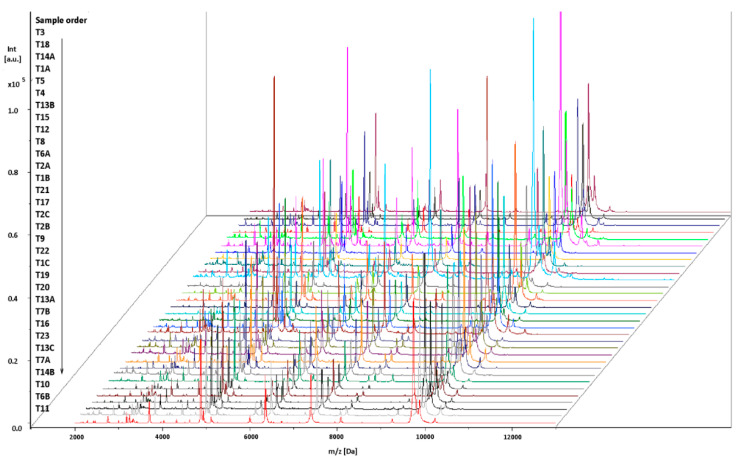
Spectra of the *L. monocytogenes* isolates generated by MALDI-TOF MS. The intensities and masses of the ions are shown on the Y- and X-axes, respectively. Sample codes displayed on the Y-axis are in accordance with those in Table 1. The m/z value is the mass-to-charge ratio.

All of the mass spectra of the tested isolates showed a good resolution, with a variety of peaks with specific protein profiles. The isolates all had 20 peaks in common, with the masses at m/z (mean peak position ± standard deviation) 2755.864 ± 0.25, 3181.643 ± 0.30, 3251.036 ± 0.23, 3304.155 ± 0.34, 3320.206 ± 0.24, 3336.243 ± 0.25, 3702.389 ± 0.30, 4044.233 ± 0.43, 4324.443 ± 0.36, 4630.144 ± 0.52, 4876.781 ± 0.38, 4942.374 ± 0.40, 5118.111 ± 0.41, 6007.757 ± 0.47, 6362.696 ± 0.50, 6421.715 ± 2.41, 8086.682 ± 0.80, 9258.724 ± 0.69, 9751.647 ± 0.85 and 9882.999 ± 0.88. These peaks can be considered characteristic of *L. monocytogenes*. Some peaks were characteristic of only some isolates. In the mass spectra of T1A, T4 and T5 isolates, there was no 4926.213 ± 1.58 peak. This peak was characteristic of the T3, T18, T14A, T8, T6A, T2A, T1B, T21, T17, T2C, T2B, T9, T22, T1C, T19, T20, T13A, T16, T7B, T7A, T14B, T10, T23, T6B, T11, T13B, T15, T12 and T13C isolates. Also, except for three isolates (T13B, T15 and T12), all isolates had a peak m/z of 9810.927 ± 0.789 (Figure 1).

### 2.2. Dendrogram for the L. monocytogenes Isolates

**Figure 2:**
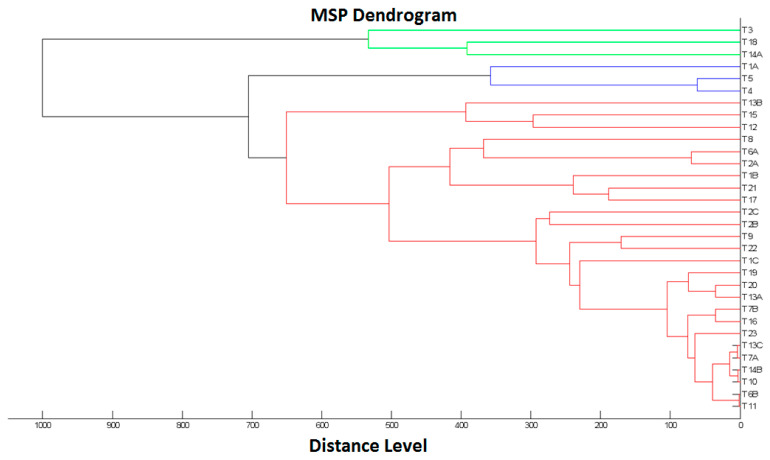
MALDI-TOF MS dendrogram showing cluster analysis of mass spectral profiles from 32 *L. monocytogenes isolates*.

The dendrogram (Figure 2) of the tested isolates indicates two main clusters. Clusters one (shown in green) and two (shown in blue and red) contained 3 and 29 isolates, respectively. Additionally, in cluster two, the isolates were grouped into three internal branches (of 3, 3 and 23 isolates), the most proteomically diverse (red) being in one of these branches.

## 3. Discussion

Ready to eat (RTE) food contaminated with *L. monocytogenes* poses a risk to public health [1]. According to current food law, the safety criterion for the products tested was the absence of *L. monocytogenes* in 25 g of the sample; therefore, 23 samples in total, i.e., 6.2% of samples of the artisanal cheeses, did not meet the food safety criterion [14]. It emerges clearly from the data presented in Table 1 that the number of cheese dairies of which the products contained *L. monocytogenes* increased between 2016 and 2018. Among them, there were new productions (e.g., G_3_, D_3_, K, H, E and B), as well as those where the pathogen was already shown in the products in previous years (e.g., I, J, C and F) (Table 1). This indicates both the need for the constant monitoring of artisanal cheeses for *L. monocytogenes* and the need for corrective action at production sites [14]. It is worth noting that, in some cases, proteomically variant isolates of *L. monocytogenes* were identified in the same sample (Figure 2, Table 1), which may indicate various co-existent sources of contamination. 

According to European Food Safety Authority (EFSA) data in the same years, i.e., 2014–2018, the frequency of *L. monocytogenes* occurrence in cheeses produced from raw or low-heat-treated milk ranged from 0.9% to 2.6% for soft and semi-soft cheeses and from 0.1% to 2.0% for hard cheeses [1,20]. Given that these data come from many European Union countries (depending on the type of cheese, data are from 9 to 22 countries), it should be concluded that the frequency of *L. monocytogenes* occurrence in the tested artisanal cheeses was higher than in other EU countries. The pathogen was more often found in traditional homemade cheeses from Turkey, such as cokelek and kumak cheese (both 30%), farm cheese (20%) [21] and white cheese (9.2%) [22], compared to the tested artisanal cheeses (6.2%). It is clear from the data presented that the frequency of *L. monocytogenes* occurrence in traditional cheeses varies, and the bacterium remains a problem concerning food safety. According to the literature, the risk of *L. monocytogenes* contamination of the final product may be due to the contamination of the raw material, milking equipment, production environment or farm workers [21,23,24,25,26,27].

In the present study, the identification of *L. monocytogenes* by MALDI-TOF MS was varied (highly probable species identification; secure genus identification, probable species identification; and probable genus identification). The vast majority of the *L. monocytogenes* isolates (27/32) were recognised to secure genus identification, probable species identification, and for the next group of isolates, as probable genus identification (4/32). Using the MALDI-TOF MS technique, it is possible to record the mass spectra reflecting the protein profiles of the analysed bacteria; however, a possibility of identification exists only for the protein profiles whose sequences are deposited in the database. Given that the analysed isolates were identified as *L. monocytogenes* by PCR, it should be concluded that the MALDI-TOF MS system database needs to be extended with spectra for strains within artisanal cheeses to increase the potential for thorough identification. Thouvenot et al. also indicated the importance of a continuously updated, high-quality reference library for the exact identification of *Listeria* [17]. Research by other authors has shown that the MALDI-TOF MS method is not without limitations in identifying *L. monocytogenes*. Pusztahelyi et al. reported that more than half of the tested bacterial strains (n = 18) were scored with values that gave secure identification only at the genus level (2.000–2.299), while only seven strains gave a highly probable species identification (>2.300). Furthermore, the same study showed the misidentification of *L. monocytogenes* at the species level [19]. In contrast, Thouvenot et al. reported the complete reliability of MALDI-TOF MS mass spectrometry to identify *Listeria* in human, animal, food and environmental microbiology, with 100% accuracy for identifying eight species, including *L. monocytogenes* [17]. The presented study results did not confirm the complete reliability of the MALDI-TOF MS method for the isolates derived from the artisanal cheeses. In view of the above, the MALDI-TOF MS method alone was not enough for a certain *L. monocytogenes* identification. As a consequence, this method was not a suitable for the routine diagnostics of *L. monocytogenes* in the foods.

In the mass spectra of the tested isolates, prominent peaks were between 2 and 11 kDa, with the highest-intensity peaks between 4 and 10 kDa (Figure 1). Studies by other authors showed that prominent peaks in the mass spectra of *L. monocytogenes* were noted in a similar range (2–10 kDa) [28].

Dendrogram analysis (Figure 2) indicated that closely related isolates, e.g., T11 and T6B, came from different administrative divisions and different cheese dairies. These were also isolated in different calendar years. Other isolates that were less related, such as T21 and T17, occurred only locally in the same administrative division and in the same cheese dairies and were also isolated in different calendar years. Therefore, the data indicated that the proteomic relationship of the *L. monocytogenes* isolates studied did not correlate with the cheese dairy, administrative division or year of isolation.

## 4. Materials and Methods

### 4.1. Materials

The research was conducted between 2014 and 2018. A total of 370 samples of artisanal cheeses were tested from cheese dairies located in Southern Poland. The cheeses were produced by traditional methods and according to long-standing recipes indigenous to the region. Advanced technological solutions were not used in the cheese production process, nor were production norms implemented (production diagram is included in Figure S1). The raw material for the production of artisanal cheeses was unpasteurised milk.

The sampling procedure was followed: a total of 5 samples of tested cheeses from each batch were taken in each of the cheese dairies [14]. The samples were taken at the producers before the cheeses were put on the market (for sale) under sterile conditions and then transferred to the laboratory at the cold-store temperature (0–4 °C).

### 4.2. Bacteriological Analysis

The presence of *L. monocytogenes* was determined according to the ISO standards [29,30]. Briefly, the mass of the sample was 25 g; selective multiplication of isolates was carried out on half-Fraser and Fraser broth; then, selective and differential media ALOA (agar *Listeria* according to Ottaviani and Agosti) and PALCAM (polymyxin acriflavine lithium chloride ceftazidime aesculin mannitol) media were used. For confirmation, 5 suspect colonies were selected from each plate of the selective and differential medium (ALOA and PALCAM), and from July 2017, from 1 to 5 suspected colonies, according to the revised procedure set out in the new version of ISO 11290-1. Material from suspect colonies was streaked on TSYEA (tryptone soya yeast extract agar), in a manner that allowed isolated colonies to develop and was then incubated at 37 °C for 24 h. The pure cultures thus obtained were tested for belonging to the *Listeria* spp. (Gram staining and catalase and bacterial motility tests) and followed by *L. monocytogenes* confirmatory tests (haemolysis test, CAMP test and the ability to decompose rhamnose and xylose). The strains thus selected were further confirmed using the MicrobactListeria 12L identification test. Each identification strip consisted of 12 tests. The following biochemical features were analysed: hydrolysis of aesculin, utilisation of specific 10 carbohydrates (mannitol, xylose, arabitol, ribose, rhamnose, trehalose, tagatose, glucose-1-phosphate and methyl-d-glucose and methyl-d-mannose), and a rapid haemolysis test was performed (micro-haemolysis). Material from confirmed pure cultures was emulsified in a suspending medium and mixed to obtain an inoculum to the MacFarland 0.5 standard. Subsequently, 4 drops of the bacterial suspension were transferred into each well. The inoculated strips were incubated at 35 ± 2 °C for 4 h. After incubation, the reactions were read visually and interpreted using the data tables provided with the test. Merck Millipore, Biomaxima and Thermo Fisher Scientific culture media were used in the bacteriological study. All isolates underwent identification using multiplex PCR. The procedure was carried out by an accredited laboratory commissioned by the Veterinary Inspectorate as part of official monitoring imposed by the applicable food law (detailed data on the molecular serotyping of *L. monocytogenes* by multiplex PCR are included in Tables S3–S6).

### 4.3. Identification of L. monocytogenes Isolates by MALDI-TOF MS

The identification of bacterial strains was preceded by the preliminary extraction of proteins with ethanol and formic acid. For this purpose, a single colony of bacterial culture was suspended in 150 µL of sterile deionised water, after which 450 µL of pure ethanol (Merck, Darmstadt, Germany) was added. Then, each sample was mixed thoroughly by vortexing. The resulting solution was then centrifuged for 5 min at 13,000 rpm. Next, the supernatant was discarded, 40 µL of 70% aqueous formic acid and 40 µL of acetonitrile (Merck, Darmstadt, Germany) were added to the precipitate and the sample was thoroughly mixed by vortexing again. After centrifugation at 13,000 rpm for 2 min, 1 µL of the obtained supernatant was applied to a metal plate and allowed to dry at room temperature. Then, 1 µL of matrix solution (cyano-4-hydroxycinnamic acid, Bruker, Bremen, Germany) was applied, and the sample was again left to dry at room temperature. The metal plate with the samples was subsequently placed in a MALDI chamber for analysis. A measurement of the spectrum and a comparative analysis with reference spectra of bacteria were performed using an UltrafleXtreme mass spectrometer at m/z range 2000–20,000 Da and MALDI Biotyper 3.1 software (Bruker Daltonik, Bremen, Germany). The results were shown as the top 10 identification matches, along with confidence scores ranging from 0.00 to 3.00. According to the criteria recommended by the manufacturer, a log (score) below 1.70 does not allow for reliable identification; a log (score) between 1.70 and 1.99 allows identification at the genus level; a log (score) between 2.00 and 2.29 indicates highly probable identification at the genus level and probable identification at the species level; and a log (score) between 2.30 and 3.00 indicates highly probable identification at the species level. Analysis of each sample was performed in triplicate, i.e., 3 spots for each sample. The identification result was considered reliable when at least the two best matches—log (score) 1.70–3.00—with the MALDI Biotyper database indicated the same species. For samples in which the top two matches indicated different species, we considered the first match—the log (score) was greater than the value for the second match.

### 4.4. Dendrogram Construction for L. monocytogenes Isolates 

A dendrogram was created by matching main spectra peaks (MSPs) of the tested *L. monocytogenes* isolates. Each MSP was matched against all MSPs of the analysed set. The list of score values was used to calculate normalised distance values between strains, resulting in a matrix of matching scores. The visualisation of the relationship between the MSPs was displayed in a dendrogram using MALDI Biotyper 3.1 software (Bruker Daltonik, Bremen, Germany) [31].

## 5. Conclusions

There is no doubt that there is a need to monitor cheeses produced by traditional methods for *L. monocytogenes*. Studies have unequivocally shown that the presence of *L. monocytogenes* in artisanal cheeses is a current problem for food safety, and these cheeses pose a potential public health risk.

The standard of species identification of *L. monocytogenes* isolates from artisanal cheeses achieved by MALDI-TOF MS was unsatisfactory, indicating the need to extend the database for mass spectra of *L. monocytogenes* strains isolated from this type of foodstuff. The MALDI-TOF MS method alone was not enough for a reliable *L. monocytogenes* identification, and as a consequence, this method cannot be considered as a suitable tool for the routine diagnostics of *L. monocytogenes* from a food safety point of view.

## Figures and Tables

**Table 1:** Identification of the *L. monocytogenes* isolates derived from the artisanal cheeses by MALDI-TOF MS.

MALDI–TOF MS.					
Production facilities	Year of sampling	Sample code	Score values	Species ID according to MALDI Biotyper 3.1	
G_1_	2014	1A	2.041–2.272	*L. monocytogenes*	DSM 20600T DSM
G_1_	2014	1B	2.028–-2.055	*L. monocytogenes*	DSM 20600T DSM
G_1_	2015	1C	2.087–2.193	*L. monocytogenes*	Mb 19348_1 CHB
D_4_	2014	16	1.803–2.036	*L. monocytogenes*	Mb 19348_1 CHB
J	2015	17	2.05–2.086	*L. monocytogenes*	Mb 19348_1 CHB
I	2015	2A	1.915–2.141	*L. monocytogenes*	DSM 20600T DSM
I	2015	2B	1.935–2.033	*L. monocytogenes*	DSM 20600T DSM
I	2015	2C	2.033–2.152	*L. monocytogenes*	Mb 19348_1 CHB
I	2016	3	1.997–2.28	*L. monocytogenes*	Mb 19348_1 CHB
D_1_	2016	4	2.239–2.29	*L. monocytogenes*	DSM 20600T DSM
D_2_	2016	5	2.052–2.191	*L. monocytogenes*	Mb 19348_1 CHB
A	2016	18	2.249–2.268	*L. monocytogenes*	Mb 19348_1 CHB
C	2016	19	1.976–1.988	*L. monocytogenes*	Mb 19348_1 CHB
G_2_	2016	6A	2.101–2.137	*L. monocytogenes*	DSM 20600T DSM
G_2_	2016	6B	1.946–2.043	*L. monocytogenes*	DSM 20600T DSM
F	2017	7A	2.042–2.122	*L. monocytogenes*	DSM 20600T DSM
F	2017	7B	2.031–2.095	*L. monocytogenes*	Mb 19348_1 CHB
J	2017	21	2.052–2.153	*L. monocytogenes*	Mb 19348_1 CHB
G_2_	2017	22	2.045–2.101	*L. monocytogenes*	Mb 19348_1 CHB
C	2017	23	1.905–2.02	*L. monocytogenes*	CCUG 315227 CCUG
F	2018	8	2.027–2.234	*L. monocytogenes*	Mb 19348_1 CHB
G_3_	2018	9	2.127–2.164	*L. monocytogenes*	Mb 19348_1 CHB
D_3_	2018	10	1.996–2.182	*L. monocytogenes*	Mb 19348_1 CHB
K	2018	12	1.901–1.929	*L. monocytogenes*	Mb 19348_1 CHB
H	2018	13A	2.023–2.048	*L. monocytogenes*	DSM 20600T DSM
H	2018	13B	1.903–2.112	*L. monocytogenes*	Mb 19348_1 CHB
H	2018	13C	1.991–2.065	*L. monocytogenes*	Mb 19348_1 CHB
I	2018	14A	2.306–2.368	*L. monocytogenes*	Mb 19348_1 CHB
I	2018	14B	2.013–2.106	*L. monocytogenes*	Mb 19348_1 CHB
I	2018	15	1.812–2.048	*L. monocytogenes*	Mb 19348_1 CHB
E	2018	20	1.922–1.955	*L. monocytogenes*	DSM 20600T DSM
B	2018	11	1.96–1.977	*L. monocytogenes*	DSM 20600T DSM

Different capital letters (from A to K) indicate the production sites in different towns, while the same capital letters with different subscripts (D_1_–D_4_, G_1_–G_3_) show different production sites in one town. Sample code marked with a number and capital letter (1A–C; 2A–C; 6A and 6B; 7A and 7B; 13A–C; 14A and 14B) shown at some production sites (G_1_, I, G_2_, F, H, I, respectively) indicate the number of isolates out of 1 tested sample.

## Data Availability

Data is contained within the article or supplementary material.

## Supplementary Materials

The following are available online at https://www.mdpi.com/article/10.3390/pathogens10060632/s1, Figure S1: The main stages in the production of artisanal cheeses; Table S1: Identification of the *L. monocytogenes* isolates by MALDI-TOF MS; Table S2: Meaning of score values; Table S3–S6: Molecular serotyping of *L. monocytogenes* by multiplex PCR.
