# Supplementary material for: Occurrence of Listeria monocytogenes in Artisanal Cheeses from Poland and Its Identification by MALDI-TOF MS
Source: Pathogens. 2021 May 21;10(6):632. doi: 10.3390/pathogens10060632 (PMC8224308; doi:10.3390/pathogens10060632)
Supplement: Supplementary file 1 [file pathogens-10-00632-s001.zip › pathogens-1204056-supplementary.pdf]

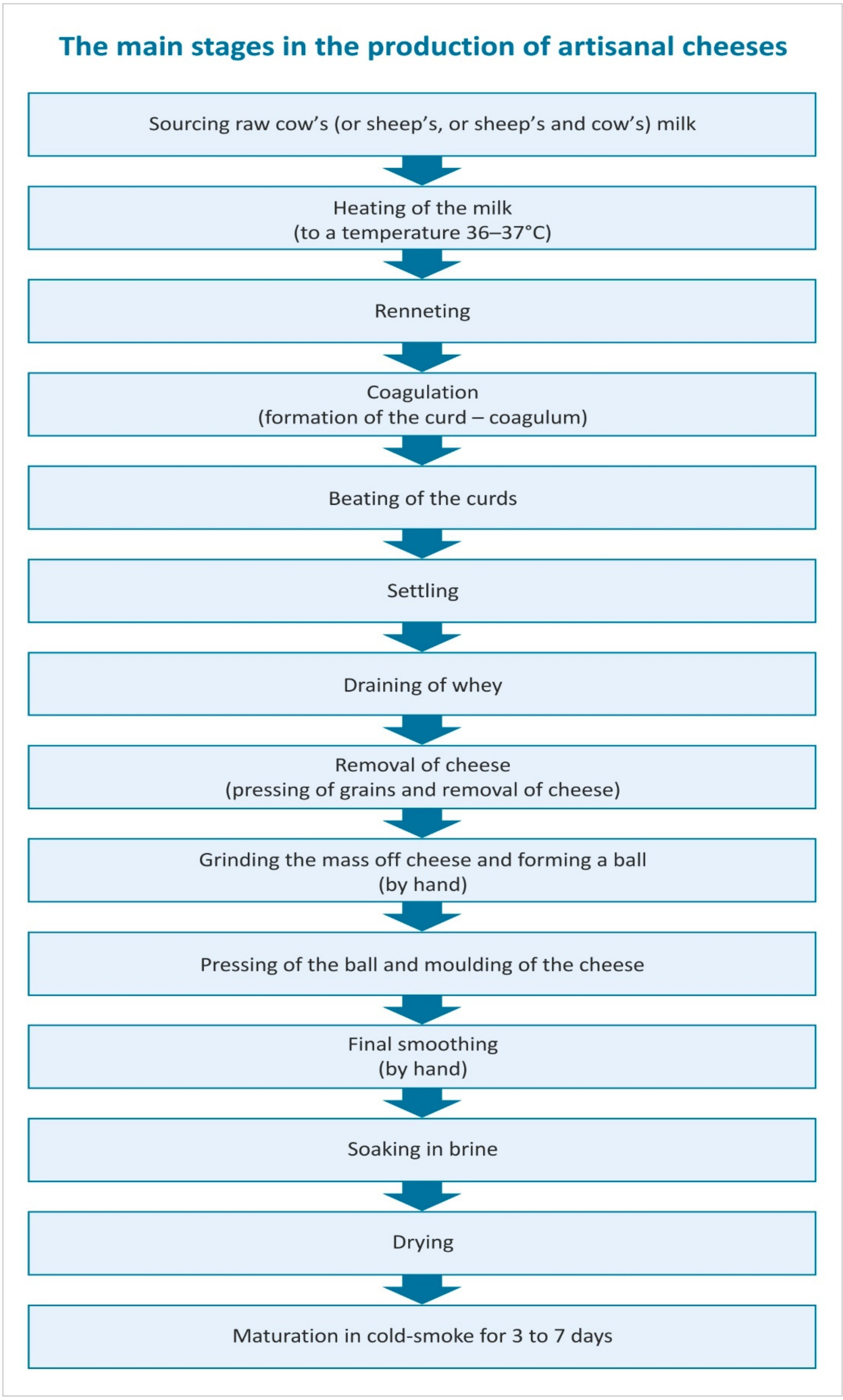

Figure S1. The main stages in the production of artisanal cheeses. The diagram contains the main stages of the artisanal cheeses production and it was compiled based on the information obtained from the producers.

MALDI TOF MS identification

Table 1. Identification of the *L. monocytogenes* isolates by MALDI TOF MS.

Analyte Name	Organism (best match)	Score Value	Organism (second best match)	Score Value
T_23_2(++) (A)	Listeria monocytogenes	2.02	Listeria monocytogenes	2.002
T_10_1(+) (B)	Listeria monocytogenes	1.996	Listeria innocua	1.996
T_11_1(+) (B)	Listeria monocytogenes	1.96	Listeria monocytogenes	1.931
T_13B_2(++) (A)	Listeria monocytogenes	2.054	Listeria monocytogenes	1.994
T_2A_1(++) (A)	Listeria monocytogenes	2.007	Listeria innocua	1.993
T_2B_2(+) (B)	Listeria monocytogenes	1.997	Listeria monocytogenes	1.991
T_6A_1(++) (B)	Listeria monocytogenes	2.101	Listeria innocua	2.023
T_16_3(++) (A)	Listeria monocytogenes	2.005	Listeria monocytogenes	1.951
T_19_2(+) (B)	Listeria monocytogenes	1.986	Listeria monocytogenes	1.955
T_4_1(++) (B)	Listeria monocytogenes	2.239	Listeria innocua	2.203
T_5_1(++) (B)	Listeria monocytogenes	2.112	Listeria monocytogenes	2.063
T_10_2(++) (B)	Listeria monocytogenes	2.182	Listeria monocytogenes	2.139
T_10_3(++) (B)	Listeria monocytogenes	2.106	Listeria monocytogenes	2.087
T_11_2(+) (B)	Listeria monocytogenes	1.997	Listeria monocytogenes	1.966
T_11_3(+) (B)	Listeria monocytogenes	1.997	Listeria monocytogenes	1.994
T_12_1(+) (B)	Listeria monocytogenes	1.921	Listeria monocytogenes	1.88
T_12_2(+) (B)	Listeria monocytogenes	1.901	Listeria welshimeri	1.834
T_12_3(+) (B)	Listeria monocytogenes	1.929	Listeria monocytogenes	1.87
T_13A_1(++) (A)	Listeria monocytogenes	2.023	Listeria monocytogenes	2.022
T_13A_2(++) (A)	Listeria monocytogenes	2.048	Listeria monocytogenes	2.023
T_13A_3(++) (A)	Listeria monocytogenes	2.024	Listeria monocytogenes	1.992
T_13B_1(+) (B)	Listeria monocytogenes	1.903	Listeria monocytogenes	1.862
T_13B_3(++) (A)	Listeria monocytogenes	2.112	Listeria monocytogenes	2.03
T_13C_1(++) (B)	Listeria monocytogenes	2.065	Listeria monocytogenes	2.049
T_13C_2(+) (B)	Listeria monocytogenes	1.991	Listeria monocytogenes	1.935
T_13C_3(++) (A)	Listeria monocytogenes	2.021	Listeria monocytogenes	1.996
T_14A_1(+++) (B)	Listeria monocytogenes	2.368	Listeria monocytogenes	2.317
T_14A_2(+++) (B)	Listeria monocytogenes	2.306	Listeria monocytogenes	2.276
T_14A_3(+++) (B)	Listeria monocytogenes	2.326	Listeria monocytogenes	2.272
T_14B_1(++) (A)	Listeria monocytogenes	2.013	Listeria monocytogenes	1.978
T_14B_2(++) (A)	Listeria monocytogenes	2.106	Listeria monocytogenes	2.017
T_14B_3(++) (A)	Listeria monocytogenes	2.106	Listeria monocytogenes	2.033
T_15_1(+) (B)	Listeria monocytogenes	1.812	Listeria monocytogenes	1.777
T_15_2(+) (B)	Listeria monocytogenes	1.951	Listeria monocytogenes	1.935
T_15_3(++) (A)	Listeria monocytogenes	2.048	Listeria monocytogenes	1.979
T_16_1(++) (A)	Listeria monocytogenes	2.036	Listeria monocytogenes	1.958
T_16_2(+) (B)	Listeria monocytogenes	1.803	Listeria monocytogenes	1.771
T_17_1(++) (B)	Listeria monocytogenes	2.05	Listeria innocua	2.048
T_17_2(++) (B)	Listeria monocytogenes	2.086	Listeria monocytogenes	2.075
T_17_3(++) (B)	Listeria monocytogenes	2.08	Listeria innocua	2.046
T_18_1(++) (B)	Listeria monocytogenes	2.268	Listeria monocytogenes	2.15
T_18_2(++) (B)	Listeria monocytogenes	2.262	Listeria monocytogenes	2.131
T_18_3(++) (B)	Listeria monocytogenes	2.249	Listeria monocytogenes	2.152

T_19_1(+) (B)	Listeria monocytogenes	1.976	Listeria ivanovii	1.967
T_19_3(+) (B)	Listeria monocytogenes	1.988	Listeria ivanovii	1.98
T_1A_1(++) (B)	Listeria monocytogenes	2.272	Listeria monocytogenes	2.213
T_1A_2(++) (B)	Listeria monocytogenes	2.258	Listeria monocytogenes	2.229
T_1A_3(++) (B)	Listeria monocytogenes	2.041	Listeria monocytogenes	2.026
T_1B_1(++) (A)	Listeria monocytogenes	2.028	Listeria monocytogenes	2.027
T_1B_2(++) (B)	Listeria monocytogenes	2.045	Listeria monocytogenes	2.019
T_1B_3(++) (B)	Listeria monocytogenes	2.055	Listeria innocua	2.05
T_1C_1(++) (B)	Listeria monocytogenes	2.169	Listeria innocua	2.154
T_1C_2(++) (B)	Listeria monocytogenes	2.193	Listeria innocua	2.139
T_1C_3(++) (B)	Listeria monocytogenes	2.087	Listeria monocytogenes	2.025
T_20_1(+) (B)	Listeria monocytogenes	1.955	Listeria monocytogenes	1.917
T_20_2(+) (B)	Listeria monocytogenes	1.922	Listeria monocytogenes	1.895
T_20_3(+) (B)	Listeria monocytogenes	1.936	Listeria monocytogenes	1.934
T_21_1(++) (B)	Listeria monocytogenes	2.116	Listeria monocytogenes	2.074
T_21_2(++) (A)	Listeria monocytogenes	2.052	Listeria monocytogenes	1.998
T_21_3(++) (B)	Listeria monocytogenes	2.153	Listeria monocytogenes	2.067
T_22_1(++) (B)	Listeria monocytogenes	2.099	Listeria monocytogenes	2.055
T_22_2(++) (A)	Listeria monocytogenes	2.101	Listeria monocytogenes	2.037
T_22_3(++) (A)	Listeria monocytogenes	2.045	Listeria monocytogenes	1.956
T_23_1(+) (B)	Listeria monocytogenes	1.905	Listeria monocytogenes	1.903
T_23_3(+) (B)	Listeria monocytogenes	1.998	Listeria welshimeri	1.943
T_2A_2(+) (B)	Listeria monocytogenes	1.915	Listeria monocytogenes	1.89
T_2A_3(++) (B)	Listeria monocytogenes	2.141	Listeria monocytogenes	2.133
T_2B_1(+) (B)	Listeria monocytogenes	1.935	Listeria monocytogenes	1.923
T_2B_3(++) (A)	Listeria monocytogenes	2.033	Listeria monocytogenes	1.985
T_2C_1(++) (A)	Listeria monocytogenes	2.061	Listeria monocytogenes	2.014
T_2C_2(++) (A)	Listeria monocytogenes	2.033	Listeria monocytogenes	1.973
T_2C_3(++) (B)	Listeria monocytogenes	2.152	Listeria monocytogenes	2.133
T_3_1(++) (B)	Listeria monocytogenes	2.28	Listeria innocua	2.28
T_3_2(++) (B)	Listeria monocytogenes	2.048	Listeria ivanovii	2.047
T_3_3(+) (B)	Listeria monocytogenes	1.997	Listeria innocua	1.961
T_4_2(++) (B)	Listeria monocytogenes	2.278	Listeria monocytogenes	2.247
T_4_3(++) (B)	Listeria monocytogenes	2.29	Listeria monocytogenes	2.225
T_5_2(++) (B)	Listeria monocytogenes	2.052	Listeria monocytogenes	2.03
T_5_3(++) (B)	Listeria monocytogenes	2.191	Listeria innocua	2.107
T_6A_2(++) (B)	Listeria monocytogenes	2.134	Listeria monocytogenes	2.091
T_6A_3(++) (B)	Listeria monocytogenes	2.137	Listeria ivanovii	2.105
T_6B_1(+) (B)	Listeria monocytogenes	1.946	Listeria welshimeri	1.865
T_6B_2(++) (A)	Listeria monocytogenes	2.043	Listeria monocytogenes	1.99
T_6B_3(+) (B)	Listeria monocytogenes	1.947	Listeria innocua	1.93
T_7A_1(++) (B)	Listeria monocytogenes	2.122	Listeria monocytogenes	2.093
T_7A_2(++) (B)	Listeria monocytogenes	2.042	Listeria monocytogenes	2.027
T_7A_3(++) (A)	Listeria monocytogenes	2.113	Listeria monocytogenes	2.062
T_7B_1(++) (A)	Listeria monocytogenes	2.065	Listeria monocytogenes	2.025
T_7B_2(++) (A)	Listeria monocytogenes	2.095	Listeria monocytogenes	2.077
T_7B_3(++) (A)	Listeria monocytogenes	2.031	Listeria monocytogenes	2

T_8_1(++) (A)	Listeria monocytogenes	2.027	Listeria monocytogenes	2.009
T_8_2(++) (B)	Listeria monocytogenes	2.234	Listeria monocytogenes	2.2
T_8_3(++) (B)	Listeria monocytogenes	2.121	Listeria monocytogenes	2.103
T_9_1(++) (B)	Listeria monocytogenes	2.144	Listeria monocytogenes	2.112
T_9_2(++) (B)	Listeria monocytogenes	2.164	Listeria monocytogenes	2.136
T_9_3(++) (B)	Listeria monocytogenes	2.127	Listeria monocytogenes	2.12

Table 2. Meaning of score values.

Range	Description	Symbols	Color
**2.300 ... 3.000**	**highly probable species identification**	**(+++)**	green
**2.000 ... 2.299**	**secure genus identification, probable species identification**	**(++)**	green
**1.700 ... 1.999**	**probable genus identification**	**(+)**	yellow
**0.000 ... 1.699**	**not reliable identification**	**(-)**	red

Molecular serotyping of *L. monocytogenes* by multiplex PCR

The Multiplex PCR for identification of the main *L. monocytogenes* serogroups was performed as described previously (Doumith et al., 2004; Wieczorek et al., 2012). The gene amplification was carried out in a thermal cycler (Biometra, G€ottingen, Germany) under the following conditions: initial DNA denaturation at 94°C for 5 min, followed by 30 cycles of 94°C for 1 min, 55°C for 1 min, and 72°C for 2 min. The final cycle was performed at 55°C for 2 min and 72°C for 5 min. The protocol developed by Doumith et al. (2014) was used to determine the molecular serogroup affiliation of the *L. monocytogenes* tested. This method facilitates assigning isolates to one of four molecular serogroups (IIa, IIb, IIc, or IVb) by the presence of the genes listed in Table S3.

Table 3. Presence of lmo0737, lmo1118, ORF2819, ORF2810 and prs in *L. monocytogenes* molecular serogroups.

**Serogroups (serotypes)**	**Presence of gen**				
	lmo118	lmo0737	ORF2110	ORF2819	prs
IIa (1/2a, 3a)		▪			▪
IIb (1/2b, 3b)				▪	▪
IIc (1/2c, 3c)	▪	▪			▪
IVb (4b,4d,4e)▪			▪	▪	▪

The reaction mixture with the constituents shown in Table S4 was prepared for the multiplex PCR, the primers for the reaction were those stated in Table S5 and the parameters of the PCR are set out in Table S6.

Table 4. Constituents of the reaction mixture in the multiplex PCR assay for *L. monocytogenes* molecular serogroup identification.

**Reaction mixture PCR (starting concentration)**	**Quantity (µl)**
water	22
bufor PCR (10x)	5
MgCl2 (25 mM)	6

dNTP (2 mM)	5
Starters (10 mM):	
lmo1118F	0,5
lmo1118R	0,5
lmo0737F	0,5
lmo0737R	0,5
ORF2110F	0,5
ORF2110R	0,5
ORF2819F	0,5
ORF2819R	0,5
prsF	0,5
prsR	0,5
Polymerase (1 U/µl)	2
DNA	5
Total	50

Table 5. Primers used in the multiplex PCR assay for *L. monocytogenes* molecular serogroup identification.

Amplified gen	Starter name	Sequence (5′→ 3′)	Product size (pz)
lmo1118	lmo1118F	AGGGGTCTTAAATCCTGGAA	906
	lmo1118R	CGGCTTGTTCGGCATACTTA	
lmo0737	lmo0737F	AGGGCTTCAAGGACTTACCC	691
	lmo0737R	ACGATTTCTGCTTGCCATTC	
ORF2110	ORF2110F	AGTGGACAATTGATTGGTGAA	597
	ORF2110R	CATCCATCCCTTACTTTGGAC	
ORF2819	ORF2819F	AGCAAAATGCCAAAACTCGT	471
	ORF2819R	CATCACTAAAGCCTCCCATTG	
prs	prsR	GCTGAAGAGATTGCGAAAGAAG	370
	prsF	CAAAGAAACCTTGGATTTGCGG	

Table 6. Multiplex PCR assay parameters for *L. monocytogenes* molecular serogroup identification.

Temperature (°C)	Number of cycles	Time (min)
94,0	1	5
55,0	30	1
72,0		2
94,0		1
55,0	1	2
72,0	1	5
15,0	∞	

After amplification, 5µl of 10× DNA gel loading buffer was added to each sample and the samples were applied to 1.5% agarose gel and electrophorosed for 40 min at 100V. The results were viewed with a GelDoc 2000 transilluminator (Bio-Rad, Hercules, CA, USA). The GeneRuler 100bp DNA Ladder (Thermo Fisher Scientific, Waltham, MA, USA) was used as the molecular weight standard.

References

1. Doumith, M.; Buchrieser, C.; Glaser, P.; Jacquet, C.; Martin, P. Differentiation of the major *Listeria monocytogenes* serovars by multiplex PCR. J. *Clin. Microbiol.* **2004**, *42*, 3819–3822. doi:10.1128/JCM.42.8.3819-3822.2004

2. Wieczorek, K.; Dmowska, K.; Osek, J. Characterization and antimicrobial resistance of *Listeria monocytogenes* isolated from retail beef meat in Poland. *Foodborne Pathog. Dis*. **2012,** *9*, 681–685. doi:10.1089/fpd.2012.1137
